# Preparation and Application of Electrodes in Capacitive Deionization (CDI): a State-of-Art Review

**DOI:** 10.1186/s11671-016-1284-1

**Published:** 2016-02-03

**Authors:** Baoping Jia, Wei Zhang

**Affiliations:** School of Materials Science and Engineering, Changzhou University, Changzhou, Jiangsu 213164 China; Centre for Water Management and Reuse, University of South Australia, Mawson Lakes, South Australia 5095 Australia; Research Centre for Water Environment Technology, Department of Urban Engineering, University of Tokyo, Tokyo, 113-0033 Japan

**Keywords:** Capacitive deionization, Electrode, Review, Desalination, Water treatment

## Abstract

As a promising desalination technology, capacitive deionization (CDI) have shown practicality and cost-effectiveness in brackish water treatment. Developing more efficient electrode materials is the key to improving salt removal performance. This work reviewed current progress on electrode fabrication in application of CDI. Fundamental principal (e.g. EDL theory and adsorption isotherms) and process factors (e.g. pore distribution, potential, salt type and concentration) of CDI performance were presented first. It was then followed by in-depth discussion and comparison on properties and fabrication technique of different electrodes, including carbon aerogel, activated carbon, carbon nanotubes, graphene and ordered mesoporous carbon. Finally, polyaniline as conductive polymer and its potential application as CDI electrode-enhancing materials were also discussed.

## Review

### Introduction

**Fig. 1:**
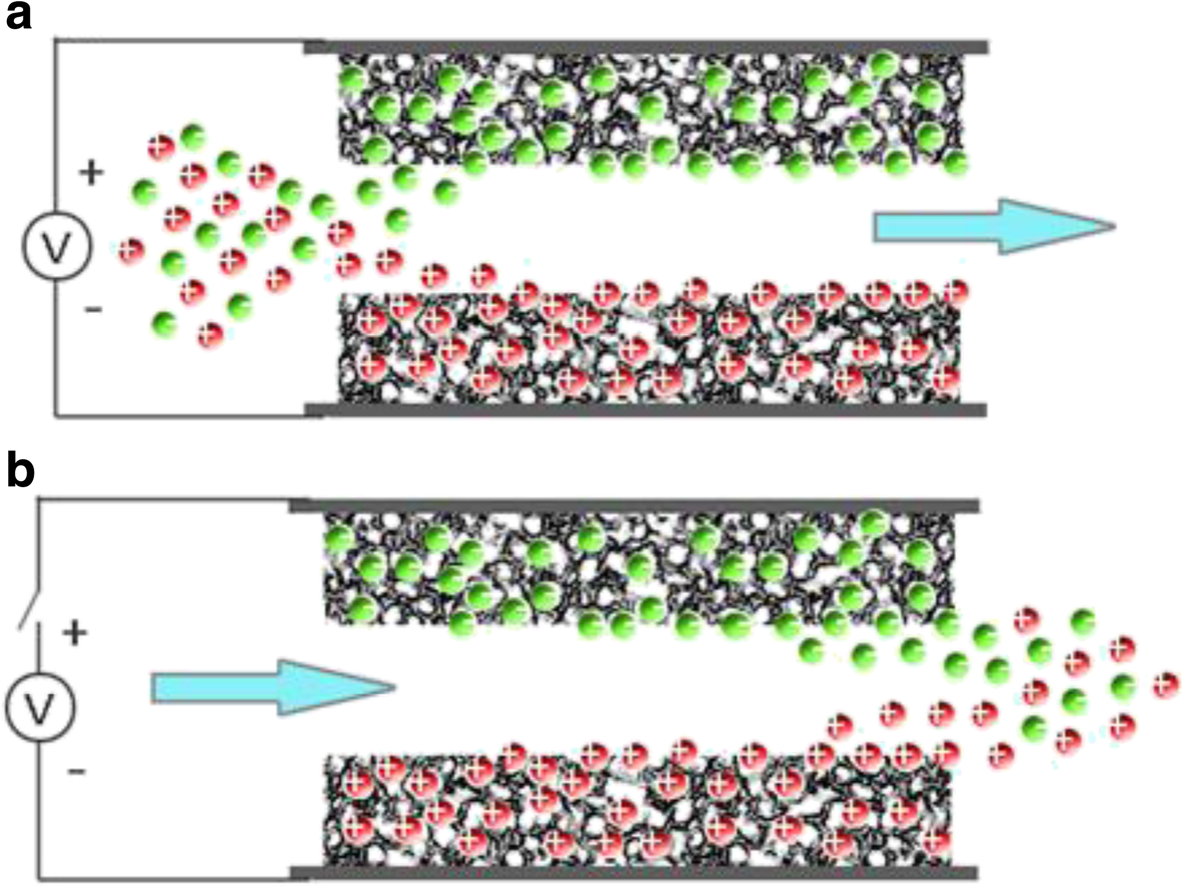
Illustration of the CDI process. **a** Ion removal procedure. **b** Electrode regeneration

The concept of capacitive deionization (CDI) was first introduced in the 1960s in the University of Oklahoma by G.W. Murphy and D.D. Caudle [1]. The first CDI system they designed was based on flew through mode in which electrode sheets were made of activated carbon powder and saline water was pumped through the charged electrode sheets. Ions were hold into the pores of the electrode sheets as a result of the static electrical force and the physical adsorption. This process was later improved by Johnson et al. [2] in the 1970s who proposed a reversible electrosorption model to regenerate the electrode by removing the electrical field of the electrode and release the ions back to a concentrate flow. Following researches included describing the process with physical theories, trying different electrode materials and designing various water flowing systems. However, the fundamental process still similar to that of Johnsons’. A typical CDI system is shown in Fig. 1.

As the ions are actually removed by the electrical adsorption and the physical adsorption effect, both the electrical double layer theory and the physical adsorption models were applied to explain the process theoretically.

#### Electrical Double Layers

Electrical double layer (EDL) is a basic concept in electrochemistry. When the electrode is charged and put into a solution with ions, the interface of the charged electrode and ions rich solution will be occupied with counter ions as a result of the Coulomb force, forming EDL. Diminishing the charge hence the removal of Coulomb force releases the held ions back to the solution. This taking up and release process can be used as deionization and regeneration operation in desalination. The first model for CDI based on electrical double layer was developed by Johnson and Newman [3, 4] in the 1970s. In this model, it was assumed that ion sorption in the CDI is approximated as capacitive process; kinetics of adsorption does not limit the rate and faradaic reactions are negligible. The model has been widely used to investigate the process and explain experimental results.

**Fig. 2:**
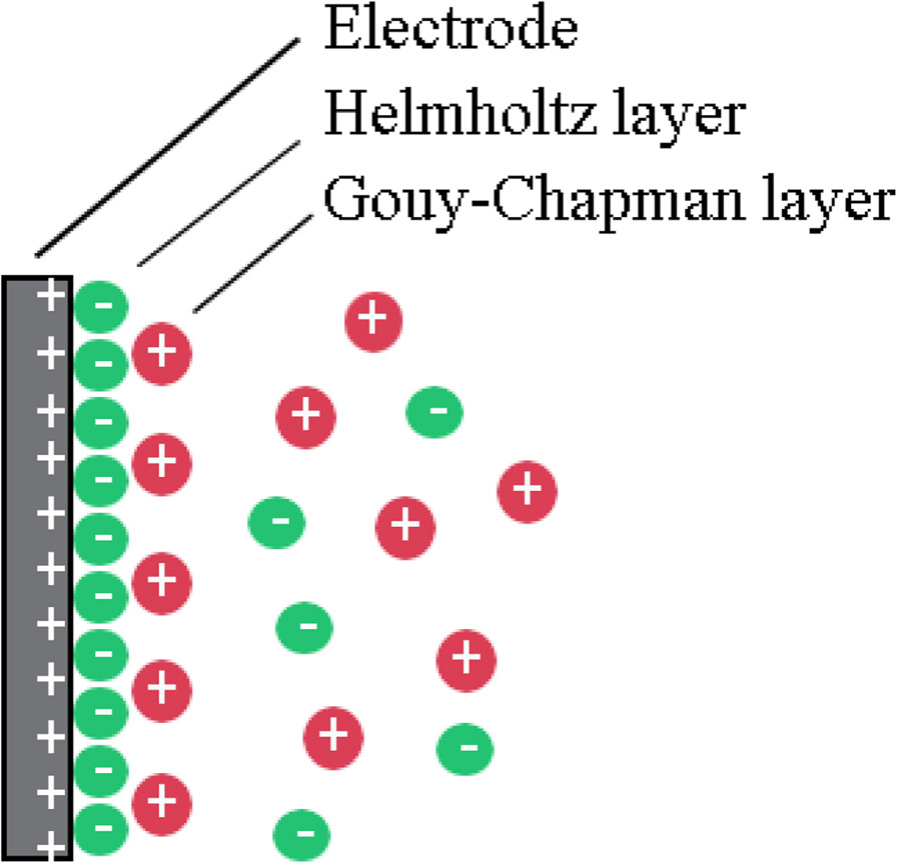
Distribution of charge in the Gouy–Chapman–Stern mode [5]

Interfacial properties and structures between an electronic conductor (electrode) and an aqueous electrolyte solution have been explained well by widely accepted Gouy-Chapman-Stern double layer theory [5]. According to the model of Gouy-Chapman-Stern model, the double layer can be assumed to be divided into an ‘inner’ region and a ‘diffusion’ region. The inner region is named as the Helmholtz layer where ions covered directly onto the surface of electrode, while the region further from the surface is a diffusion layer called the Gouy–Chapman layer where the distribution of electric charge is depended on the potential at the surface. Both capacitances of the two layers contribute to the total capacitance; thus, the total capacitance can be calculated like a series union of both the inner Helmholtz layer and diffuse Gouy–Chapman layer (Fig. 2). The electrical capacitance of the interface, *C*_*T*_, is expressed as the sum of two capacitors in series: Eq. (1).

1 $$ \frac{1}{C_T}=\frac{1}{C_{M-H}}+\frac{1}{C_{H-S}} $$

where *C*_*M−H*_ is the capacity of the inner double layer between the electrode surface *M* and the plane of closest approach for the ions *H* (Helmholtz layer, and *C*_*H−S*_ is the capacity of the Gouy-Chapman layer).

In a classic parallel-plate capacitor, charge separation is electrostatic. Capacitance is in proportion directly with the area of the plates and the inverse distance of separation as shown in Eq. (2):

2 $$ {C}_{M-H}={\varepsilon}_r{\varepsilon}_0\frac{A}{D} $$

where *C*_*M−H*_ is the capacitance (*F*); *A* is the area of each plate (*m*^2^); *ε*_*r*_ is the relative electro static permittivity (dielectric constant) of the material between the plates; *ε*_0_ is the permittivity of free space (8.854 × 10^−12^ F/m) and *D* is the separation between the plates (*m*);

Gouy and Chapman developed the formation for calculating the dependence of the excess charge, *q* on the potential at the Helmholtz plane (Eq. 3), by treating ions as point charges and assuming an ideally polarized electrode, namely, no electron transfer through the interface and ignoring the ion adsorption associated with other interactions.

3 $$ q={\left({\scriptscriptstyle \frac{2RT\varepsilon {C}_s}{\pi }}\right)}^{1/2} \sinh \left({\scriptscriptstyle \frac{z\mathrm{\Im}{\Phi}_H}{2RT}}\right) $$

Differentiating the charge q with respect to *Φ*_*H*_ the electrical capacity of the diffuse layer is obtained by Eq. (4):

4 $$ {C}_{H-S}=\left|z\right|\mathrm{\Im}{\left({\scriptscriptstyle \frac{\mathrm{\mathcal{E}}{C}_s}{2RT\pi }}\right)}^{1/2} \cosh \left({\scriptscriptstyle \frac{\left|z\right|\mathrm{\Im}{\Phi}_H}{2RT}}\right) $$

The excess charge distribution at the interface between a charged electrode-electrolyte according to the Gouy–Chapman–Stern model is described schematically in Fig. 2.

From Eq. (1), it is shown that both the capacity of the inner Helmholtz layer, *C*_*M−H*_, and the capacity of the Gouy–Chapman layer, *C*_*H−S*_, contribute to the total capacitance. However, in practical CDI processes, *C*_*M−H*_ is much more important for ions capacity. Equation (2) shows that the specific electrical capacities can reach high values, as a result of the extremely short distances involved in double layer capacitors, large relative electrostatic permittivity of the material between the plates and large specific surface of the electrodes. In fact, distances involved in double layer structure have been reduced to an extreme, and the permittivity of the material between the plates can hardly improve because of the aqueous environment in CDI cells. The option of increasing the specific surface area of the electrodes becomes the main solution to achieve high electrical capacity.

Biesheuvel et al. [6–8] further studied the Gouy–Chapman–Stern-based adsorption and desorption model in CDI process. They took a simplified process by neglecting the effect of concentration gradients and consider only the driving force of electric field. In calculation, they found only the Helmholtz layer capacity was a freely adjustable parameter that described the data well suggesting that the Helmholtz layer capacity was of equal importance to surface area in characterization of salt storage capacity. Also, taking the microporous structure of the electrode material into account, they tried to evaluate the influence of co-ion expulsion by defining charge efficiency as the ratio between the amount of salt molecules removed from the solution and the amount of electronic charge transferred between the electrodes. Charge efficiency was used as a factor to evaluate ion distribution within the electrical double layer. It was also used as indicator for estimation of energy efficiency [9–11].

#### Adsorption Model

##### Langmuir Isotherm

In traditional water treatment, carbon was used as adsorbent to removal impurities from water by an adsorption process without the help of driving force of electrical field. Several models have been used to describe the adsorption process, and Langmuir and Freundlich adsorption isotherms are the most commonly used ones. CDI is a similar process in which electrical force driving is used instead of driving of concentration gradient. In both cases, equilibrium happens as a result of the surface saturation of the carbon material. Adsorption models that have been used to studied carbon adsorption are also used to study CDI process. Although electrical double layer theory is the most commonly used model for CDI, Jayson et al. [12] reported the adsorption of mercury(II) acetate from aqueous solution onto an activated charcoal cloth (ACC) was Langmuir charactered. Gabelich et al. [13] used Langmuir, Freundlich and BET isotherms in their study on electrosorption of ions onto carbon aerogels to fit experimental data and found the Langmuir isotherm described the data well. Their findings suggested that for modelling purpose in CDI, monolayer adsorption could be assumed. The Langmuir adsorption isotherm is expressed as

5 $$ X={\scriptscriptstyle \frac{X_mb{C}_e}{1+b{C}_e}} $$

where *X* per unit weight of adsorbent; *C*_*e*_ represents equilibrium concentration of the solute.

##### Freundlich Isotherm

The Freundlich isotherm is an empirical mathematical description of adsorption in aqueous system, as

6 $$ {\scriptscriptstyle \frac{x}{m}}=K{C}_e^{1/n} $$

where *x* is the amount of solute adsorbed; *m* refers to the weight of adsorbent; *C*_*e*_ represents equilibrium concentration of the solute; and *K* and *n* are constants depending on the system.

Li et al. [14, 15] studied the adsorption isotherm of NaCl on carbon nanotube and carbon nanofibre composite. By comparing the Langmuir and Freundlich isotherms, they found the Langmuir isotherm described the experimental data better than Freundlich isotherm. Interestingly, their following studies [16, 17] on electrosorption of carbon nanotubes and graphene showed that at low salt concentration, the electrosorption followed Freundlich isotherm, too. Explanation for this phenomenon was the adsorbed layer did not fulfil the monolayer capacity at diluted solution. At this point, the Freundlich isotherm as an empirical equation of adsorption described the data well. Ban et al. [18] on the other hand found the electrosorption of naphthalenesulfonic acid on activated carbon fits the Freundlich isotherm.

### Parameters That Affect the CDI Performance

Many factors influence the adsorptive performance of the electrode material: first, the nature of the electrode material such as surface area, pore size and distribution; second, applied electrical potential and third, the solution conditions, concentration and type of ions. Gabelich et al. [19] carefully studied the effects of operational parameters including applied voltage, flow rate and pH on the system performance for the first time in a carbon aerogel-based CDI device. Obviously, all the parameters affect ion sorption and desorption process; salt removal was better at higher applied voltages (2.0 V in their study); increased sorption efficiency was obtained at slower flow rates (50 mL/min) and high pH did adversely affect desorption. Further studies addressing the determining factor for CDI found that pore distribution of the electrode material and drive potential as well as ion type and concentration influence the deionization performance significantly.

#### Pore Distribution

Early in Cauddle and Farmer’s work, it was found that the average pore size of the electrode material had significant effect on the ion removal performance. Yang et al. [20] conducted special work on the relationship between electrosorption behaviour and the pore size of carbon. They took the overlapping effect into the model by defining the cut-off pore size as the smallest width that contribute to the electrical double layers. The cut-off pore width for 100 ppm NaF at the voltage of 1.2 V is 0.6 nm. Any pore with a width smaller than 0.6 nm does not hold any ion. Mesosized pores can provide a smaller resistance for the ion transport pathway through the porous carbon [21] and also a high surface area for the ion adsorption [22]. When pore size is larger than 10 nm, the amount of ions adsorbed remain constant. For the regions between 0.6 and 10 nm, the electrical double layers overlap to some extent resulting in partially losing the anion capacity. Macrosized pores can serve as ion-buffering reservoirs guarantees shorter ion diffusion distance, which facilitates the rapid transportation of the ions into the interior of the bulk material [23]. Noked et al. [11] carried out experiments of ions of different dimensions adsorbed onto carbon electrodes with various porous structures. They found that the ratio between the pore size and the ion size affected the electrosorption process significantly. When the ions’ radii approached the pore dimension, the electrical double layer charging process was impeded. They also reported that the rate of double layer charging was not only depended on high capacitance. Lager pore size leads to faster adsorption rate. The author suggested that multiporous fractal-structured electrodes with mesopores being the entries of micropores could be a better choice that facilitates both high capacitance and faster adsorption. Peng et al. [24] compared the electrosorption behaviour of mesoporous carbon electrodes with various pore structures and found the electrode of 2D hexagonal space groups showed better ion removal performance for monovalent cation solutions, while 3D symmetry cubic carbon was more efficient in removal of large-sized bivalent and trivalent valent ions.

#### Potential

Farmer et al. [25, 26] first studied the NaCl removal with four different voltages of 0.6, 0.8, 1.0 and 1.2. They found with the increase of applying voltage the ion removal efficiency improved significantly. Similar results also found in deionization of NH_4_ClO_4_. It is easy to interpret these results from the view of EDL. Higher voltage means more free electric charge on the electrode surfaces. However, to avoid the electrical dialysis of water, the voltage applied must have an upper limit. The standard potential of the water electrolysis is 1.23 V [5], so most researchers conduct the experiment at the voltage of 1.2 V. Lee et al. [27] investigated the electrode reactions related to the applied potential by monitoring the pH value of the solution during adsorption/desorption process. It was found that pH changed correspondingly to the applied potential.

7 $$ \mathrm{H}{{\mathrm{O}}_2}^{- } + 2{\mathrm{H}}_2\mathrm{O} + 2{\mathrm{e}}^{- }\ \to\ 3\mathrm{O}{\mathrm{H}}^{-}\kern0.5em \left(E{}^{\circ}=0.87\mathrm{V}\right) $$

8 $$ 2\mathrm{C}{\mathrm{l}}^{-}\to\ \mathrm{C}{\mathrm{l}}_2\left(\mathrm{a}\mathrm{q}\right) + 2{\mathrm{e}}^{-}\kern1.25em \left(\mathrm{E}{}^{\circ}=1.39\ \mathrm{V}\right) $$

The solution pH increased at a potential of 1.0 V due to the reduction of dissolved oxygen and decreased rapidly at potentials of 1.2 V or greater due to the oxidation of chloride at the anode. The pH level of the solution was around 10 when the adsorption equilibrium was achieved while applying voltage of 0.8 and 1.0 V. When applying a potential of 1.2 V, pH increases to 10 and drops to less than 8. With 1.2 V potential, it increases to 9 and drop quickly below 4.

#### Salt Type and Concentration

In Johnson’s study [3] on electrosorption of activated carbon powders, it was observed that divalent ions were preferred to adsorb onto carbon surface than monovalent ions. The preferential sorption of divalent ions was attributed to chemical equilibrium in the electrical double layers. Gabelich et al. [13] later studied the effects of ion charge and size on sorption onto carbon aerogel electrode specifically. Results contrary to Jonson’s observation were found. Adsorption of single-valent ions was prior to divalent species when both ionic species were present. But capacity of divalent ions could be enhanced when both ions were divalent. The author suspected hydrated radius of ions dictated the process. Seo et al. [28] discussed this phenomena and gave an acceptable explanation. They considered the two contrary results were obtained using different electrode material. Differences in pore structure of the two materials caused the difference in adsorption properties. Smaller size of pores attributed to the selective adsorption of monovalent ions which are small in hydrated radius while larger pore size favoured divalent ions with large hydrated radius. Noked et al. [29] took further investigation on this problem. They proposed the ratio between pore size of electrode and hydrated ion size and demonstrated the ratio were of crucial effect in the electrosorption process. When the ions’ hydrated radius approached pore dimension of carbon, the EDL charging was impeded. The ionic strength which determines the effective diameter of ions was considered to be another important factor in electrosorption, as high ionic strength results in smaller hydration shells leading to easier entrance of ions. Furthermore, salt concentration was taken into consideration. The salt concentration influenced the electrosorption process in two ways. One way was by determining the resistance of the solution. Lower concentration means higher resistance hence slower kinetics. Another way was through ionic strength and effective size.

### Development of Electrode Materials in CDI

#### Carbon Material

The first CDI system in Caudle’s [1] research was a flew through system with electrode sheets made of activated carbon powder. Then, in the 1970s, Johnson et al. [2] continued the study and developed the process as a reversible electrosorption. Several carbon materials were studied as electrode material in their work. A theoretical model based on the electrical double layer theory was first proposed. Apparently, the electrode material should be of large specific surface area to have high capacity to hold considerable quantity of ions and be physically stable during the ions taking and release procedure to avoid polluting the water flew and gain good regeneration property. At the same time, in order to complete the procedure quickly, the electrode should be of high electrical conductivity. Porous carbon due to its high surface area, good electrical conductivity as well as low cost was used as electrode in this electrochemical deionization process. Various carbon materials including activated carbon powder, carbon aerogel, carbon nanotubes and graphene for instance are studied as candidates of electrode materials for CDI.

Carbon material is a large family represents a wide range of materials that are mainly composed by element carbon. Among them, graphite and diamond are the two with crystal structures and are not property adsorption materials because of the lack of surface area. Others are normally referred to as amorphous carbon. Characterization of these amorphous carbon shows they also have microcrystalline structure. Unlike graphite in which flat layers of hexagonal carbon rings compact together to give a crystalline structure, the orientation of microcrystalline layers in amorphous carbon are different and less ordered. The random arranged disordered microcrystallites tend to crosslink with each other which result in a well-developed porous structure hence enormous internal surface [18]. These high surface porous-structured carbons are known as activated carbons and have been widely used as adsorbents for odour, colour and organic impurities for a long time because of their unique porous structure [30]. Even before CDI, carbon had already been used as adsorbent in water treatment. With the highly favourable structure, it is no coincident that carbon is considered to be the most promising electrode material in CDI.

Apart from activated carbon, there are other carbon materials structured with carbon atoms in certain patterns such as tubes (carbon nanotubes) and sheets (graphene). These structure patterns provide the materials with unique properties, such as large surface area, high electrical conductivity and good thermal conductivity. Applications of these materials in CDI will be discussed in detail later in this part.

##### Activated Carbon

Activated carbon powder (AC) as a strong adsorbent in industry application is mainly produced by the pyrolysis of carbonaceous source materials such as nutshells and wood at temperatures lower than 1000 °C. The raw carbon materials are first carbonized at temperatures below 800 °C and followed by the activation of carbonized product between the temperatures of 950 and 1000 °C [30]. Alternatively, the raw materials could be chemically activated by acid, strong base or salt prior to carbonization and then carbonized at lower temperature (400–700 °C) [31].

AC has been the mostly used electrode materials in CDI mainly because its low cost. It was actually the first electrode materials used for electrosorption. The history of AC as an electrode material in capacitive desalination date back to the early 1960s by Caudle and Murphy [1, 32]. Since then, AC has been the most common material for CDI electrode. Normally, a CDI electrode was fabricated by a certain ratio of carbon material, conductive additives (graphite in most case) and binders. Research works on AC in CDI include modifying the pore structure, retaining faradaic reaction determining the type and amount of binder and improving the conductive property. Zou et al. [33, 34], Lee et al. [27, 35], Choi et al. [36, 37], Chang et al. [38] and Hou et al. [39] published paper on AC electrode CDI, Zou et al. emphasized on the pore structure optimization, Lee investigated the effect of operation parameter of the system, Choi et al. worked on controlling the faradaic reaction of the electrode by using poly(vinylidene fluoride) binder, Chang et al. obtained promising AC electrode by using a novel liquid binder and Hou et al. estimated that the suitable amount of binder (PVDF in their study) was 10 %. Kim et al. [40] investigated the desalination of brackish water containing oil compound using AC electrode and found the process was reproducible adsorption and desorption characteristic. Bouhadana et al. [41, 42] studied the practical aspects of an AC-based CDI system, in which the author designed a flow-by system. By estimating the energy consuming of the system, they concluded that cost-effective production of drinking water is possible by CDI process. Avraham et al. [9, 10, 43] studied the limitations of charge efficiency of the AC electrode in CDI process, in which electrosorption behaviour of a single AC electrode and double AC electrodes and the surface oxidized AC electrode were well studied. The author concluded that it is possible to maximize the charge efficiency by applying discharge potentials (upon regeneration) higher than zero and for AC of pore-size larger than 0.58 nm, the oxygen-containing surface groups have no obvious effect in the ion adsorption/desorption process.

Recently, modifying the AC electrode was used as new strategy to obtain better desalination performance. AC electrode with deposited ion-selective membrane [44], making AC electrode with water soluble blinder [45], and compositing AC with other materials to enhance its conductivity, improve the capacity and modify its pore structure have been reported. In the case of combining AC with other materials, candidate such as ion-exchange resin [35], MnO_2_ [46], titania [47] and graphene [48] were used as component to improve capacitance and desalination performance of activated carbon powder. The adding to ion-exchange resin to the electrode improved the hydrophilicity of electrode, and the ion exchange site enhanced the ion removal capacity. Deionization performance of the ion-exchange resin/AC electrode was enhanced by 35 % compared to the one without ion-exchange resin. The MnO_2_/AC composite showed doubled ion removal amount than the AC material due to a more suitable pore size distribution and effective cation intercalation of MnO_2_. For titania-loaded AC, the formation rate of electrical double layer in AC electrode was improved significantly and the amount of ions removed was increased by 62.7 %, along with good regeneration performance of electrode. Graphene-containing AC showed improved ion capacity as the graphene sheets acted as spacer that prevented the aggregation of AC, providing more available surface.

##### Carbon Aerogel

**Fig. 3:**
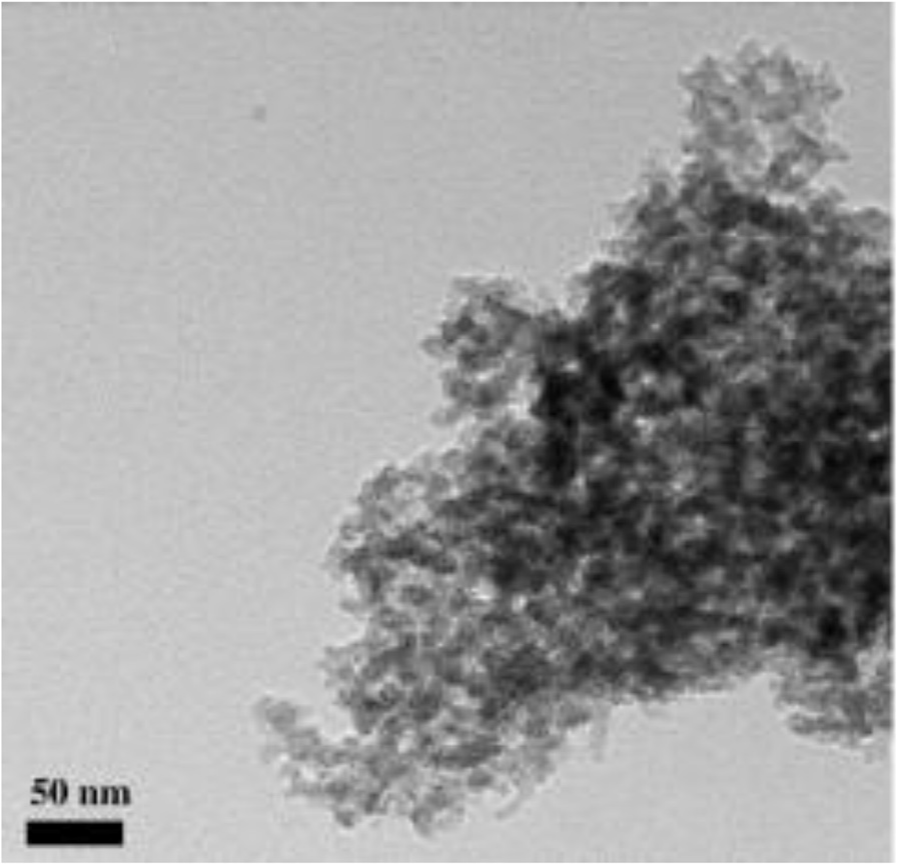
TEM image of carbon aerogels [53]

Carbon aerogels are nanosize air-filled foams with mainly carbon as skeleton. Synthesis of carbon aerogel starts by sol-gel polymerization of resorcinol (1,3-dihydroxy benzene) with formaldehyde. Polycondensation of resorcinol and formaldehyde form highly cross-linked transparent hydrogels which are dried under supercritical condition to form the precursor called RF aerogel. Pyrolyzing the RF aerogel at relatively high temperature (600–1200 °C) in an inert atmosphere gives carbon final product aerogel. Other organic aerogels, phenol-furfural, phenol-resorcinol-formaldehyde, melamine-formaldehyde, polyurethanes, polyureas and polyvinyl chloride for instance can also be used as precursors [49, 50]. Carbon aerogels are attractive as electrode materials because of their exceptional property such as high surface area (400–1000 m^2^/g) and good electrical conductivity (10–100 S/cm) [51]. Also, morphology of carbon aerogels can be controlled to be films, powders or microspheres [52]. Figure 3 shows the TEM image of carbon aerogels.

In the 1990s, carbon aerogel was synthesized and became a promising candidate for energy storage and CDI electrode material. Farmer et al. [25, 26, 54–58] from Lawrence Livermore National Laboratory did a lot of work on the application of carbon aerogel in CDI. Salts such as NaCl, NaNO_3_, Na_3_PO_4_ · Na_2_SO_4_, NH_4_ClO_4_ and hexavalent chromium were shown to be removed from aqueous solution. Since carbon aerogel could be produced as monolithic sheets by controlling the morphology, Farmer developed electrodes with pairs of thin sheets of carbon aerogels instead of deep bed. The electrolyte flowed in a channel between anodes and cathodes so as not to create a potential drop as it did in flowing though packed beds thus avoiding entraining the electrode material into the fluid stream. Their experiments showed that after prolonged operation, the electrodes lost part of their adsorption capacity, but it is believed that reversing the electrodes could make recover most of the losses. Later in 2002, Gabelich et al. [13] conducted a thorough study on the electrosorption of ions on carbon aerogels including effect of ionic charge, radius and mass. It was found that monovalent ions with smaller hydrated radii were preferentially removed from the solutions over multivalent ions and because of the small pore size only a few percentage (14–42 m^2^/g) of the BET electrode surface area (400–1000 m^2^/g) were available for electrosorption. Fouling of aerogel surface by nature organic matter was deemed to be a problem for the effective treatment of nature water. Yang et al. [59] tried to use silica gel enforced carbon aerogel as CDI electrodes. Results of their experiments showed the adding silica improved ion capacity by up to 28 % due to increased wettability and enhanced mechanical hardness.

##### OMCs

**Fig. 4:**
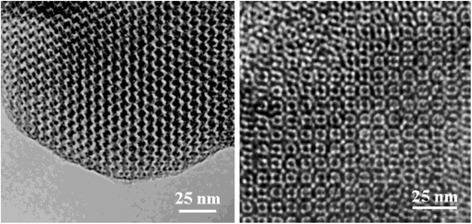
SEM images of OMC [61]

Ordered mesoporous carbons (OMCs) are carbon materials with ordered uniform pore size in the range of 2–50 nm. Typical SEM images of OMC are shown in Fig. 4. Conventionally, the uniform pore structures are obtained by attaching carbon precursors onto certain templates with three-dimensional pore structures (e.g. silica and zeolite) and remove the template after carbonization. This is called hard-template method. To overcome time-consuming and costly steps of the silica etching process, a soft-templating approach was introduced by directly assembly of organic amphiphilic surfactant or block copolymers templates with the carbon precursors [60].

Zou et al. [62–64] first studied the electrosorption performance of OMC and compared with AC electrode. They found the ion removal performance of OMC was much better than AC due to its optimized pore structure. The ordered uniform structure with a peak diameter of 4.0 nm facilitated the fast entrance and release of ions onto and off the electrode surface lead to high ion removal and faster regeneration. They further modified the OMC structure by introducing nickel salts during the synthesis process. The nickel containing OMC had smaller ordered mesopores; the peak diameter shifted to 3.7 nm from the original 4.0 nm. Also, the modified OMC possessed higher surface area and exhibited higher ion capacity. Tsouris et al. [65] compared the deionization performance of self-assembled OMC with carbon aerogel and reported that OMC was a more suitable choice for CDI. The reason was attributed to the order pore size which provided more surface area to be accessed by ions. Nadakatti et al. [66] also reported the enhancement of CDI performance by adding mesoporous carbon black. It is demonstrated that the ion removal efficiency increased by 100 % after adding the mesoporous carbon black. More recently, Peng et al. [67] synthesized composites of OMC and carbon nanotube for CDI. They found that with 10 wt.% carbon nanotube combined, the composite exhibited higher ion removal efficiency. Meanwhile, reversibility and stability of the composite electrode were both improved.

##### CNT and Graphene

**Fig. 5:**
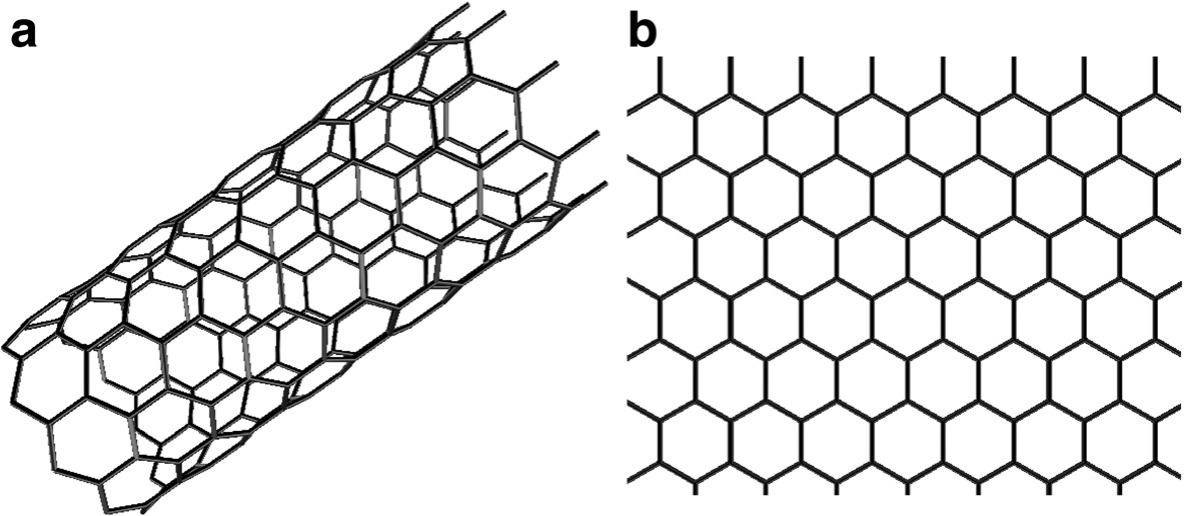
Structural illustration of carbon nanotube (**a**) and graphene (**b**)

Graphene and carbon nanotube (CNT) are novel carbon materials with unique structure and extraordinary electrical properties. Graphene is one-layered graphite in which a one-atom-thick sheet is formed by carbon atoms arranged in a regular hexagonal pattern (Fig. 5b). It is a two-dimensional material in which carbon atoms are sp^2^-bonded in the bond length of 0.144 nm. The electron mobility of graphene can be as high as 200,000 cm^2^V^−1^s^−1^ [68]. This demonstrates that the electrical conductivity of graphene is excellent. More importantly, being a single-atom layer structure, the theoretical surface area of graphene is 2630 m^2^/g [69]. Besides, the interlayered open structure of graphene sheets favours easy ion adsorption and desorption. A single-walled carbon nanotube (SWCNT) is a rolled graphene sheet in a cylindrical shape (Fig. 5a). A tube surrounding tube structure made of two layers of graphene sheets is called double-walled carbon nanotube (DWCNT). And the one made of more than two layers of graphene sheets is called multi-walled carbon nanotube (MWCNT). Depending on the arrangement of the hexagon rings along the tubular surface, CNT can be metallic or semiconducting.

These properties of uniform structure, large surface area and good conductivity make graphene and CNT very promising for a CDI electrode. Since ions are electrically adsorbed and stored in the EDL on the electrode surface, higher electrical conductivity and larger specific surface areas mean higher ion capacity [70, 71]. Compared to other carbon materials, CNT and graphene not only possess high specific surface area but also are excellent in electrical conductivity. Good conductivity not only contributes to the ion capacity but also speeds up the ion adsorption and desorption process.

Dai et al. [72, 73] reported the use of multi-walled carbon nanotube-based electrodes in the adsorption of NaCl and confirmed the promising properties of carbon nanotubes in the CDI process. Li and co-workers [15, 74] using carbon nanotubes and carbon nanofiber composites as CDI electrode material, they compared the electrosorptive properties of SWCNTs and DWCNTs, and the results of these studies are promising with respect to the suitability of CNTs as electrode material. Zhang et al. studied the effect of CNT diameter on its performance as CDI electrode [75]. They discovered that salt removal efficiency increase with the decrease of CNT diameter due to its enhanced surface area.

Pan et al. [14, 76, 77] did a great deal of work on the application of carbon nanotubes and graphene. They studied the effects of ion charge, size and mass on electrosorption of the ions. The result showed better electrosorption capacity was obtained for the anions with smaller hydrate radius. Capacity pattern of Cl^−^ > NO_3_^−^ > SO_4_^2−^ was found as smaller ions has easier access to the porous network. Meanwhile, the carbon nanotubes exhibited quite efficient regeneration performance due to high electrical conductivity. Wang et al. [78] prepared three-dimensional continuous carbon nanotube sponge by chemical vapour deposition and studied its capacitive deionization performance. The carbon nanotube sponge electrodes showed higher desalination capacity than that of AC, AC fibre and carbon aerogel. Composites of carbon nanotube with mesoporous carbon [67, 79], polyacrylic acid [80] and graphene [81, 82] were also used as electrode material in CDI. The mesoporous carbon/carbon nanotube composite exhibited excellent desalination behaviour with low energy consumption. Carbon nanotube/polyacrylic acid composite film showed higher ion capacity than pure CNT with good regeneration ability. Graphene/carbon nanotube composite demonstrated significant synergistic effect as the ion capacity of the composite was better that either of the component itself. It was explained that the long CNTs not only interconnect adjacent graphene sheets thus increasing conductivity of the composite but also inhibited the aggregation of individual sheets, providing more applicable surface.

Graphene as the novel material in CDI research showed good deionization performance than activated carbon powder, even with a much lower surface area. Li et al. [16, 17, 83] first reported the electrosorption behaviour of graphene synthesized by a modified Hummers method in NaCl solution. They later studied the ferric ion removal of the graphene material. Inspired by these research, Wang et al. [84, 85] conducted research on capacitive deionization performance of resol functionalized graphene, in which the resol restricted the aggregation of graphene resulting in higher ion capacity. Jia et al. [86] developed a three-step reduction of graphene oxide (GO) in which the GO was first mildly reduced by iron powder and then sulphonic functional groups were introduced to the partially reduced GO to obtain good dispersion property. The functionalized GO was finally reduced by hydrazine. In this way, the resultant graphene electrode showed high ion capacity and good regeneration performance. Wang et al. reported a modified thermal reduction method utilizing pyridine as intercalating agent to prepare graphene for CDI application [87]. Lei et al. prepared graphene nanosheets by a novel Fe-catalyzed carbonization method using glucose as carbon source [88]. More recently, graphene featuring three-dimensional structure as high performance CDI electrode using different templating methods was demonstrated due to higher surface area, pore volume and superior ion transportation pathway [89, 90]. Possibility of graphene/OMC composites as CDI electrode using core-shell and triblock-copolymer templating method was also explored [91, 92].

##### Other Carbon Materials

Activated carbon cloth (ACC) is another form of carbon material that has been well studied for CDI. Ryoo et al. [93, 94] carried out electrosorption experiments on ACC and silicon, aluminium and titania modified ACC. It was found that titania could be highly dispersed on ACC surface hence reduce its polar group, resulting in a reduction of physical adsorption. While electrosorption of titania modified, ACC was enhanced significantly due to increase of adsorption sites introduced by the incorporated titania atoms. Rapid desorption and good reversibility was also obtained with titania modification. Oh et al. [95] studied the influence of chemical activation of ACC on its deionization property. Myint et al. studied the CDI electrode made of nano/microsized zinc oxide/ACC composites [96], where up to 22 % salt removal efficiency was observed compared with using ACC alone.

A pair of asymmetric electrodes made of nanoporous silicon dioxide and magnesium-doped aluminium oxide was synthesized and examined for CDI application by Leonard et al. [97]. It was reported that the electrodes pair demonstrated effective removal of cations with various valences as well as decreased regeneration time. Haro et al. [98] developed carbon gel to remove ions from brackish water. Parada et al. [99] reported carbide-derived carbon for CDI and indicated that it could be a good choice as electrode material for CDI based on easy controlling of pore size. Yang et al. [100] prepared a MnO_2_/PSS/CNTs composite electrodes, and Liu et al. [101] studied electrosorption of polypyrrole and graphite composites. More recently, CDI performance of hollow carbon nanofibers, which was prepared by the electrospinning process of polyacrylonitrile (shell) and poly(methyl methacrylate) (core) [102], activated carbon fibre [103], and three-dimensional hierarchical porous carbon (3DHPC) [104] was also reported in the literature. Table 1 summarizes the CDI performances of different electrode materials.

**Table 1:** CDI performances of various electrode materials

Electrodes	Specific surface area (m^2^/g)	Initial conductivity (μS/cm)	Initial concentration (mg/L)	Percent of salt removed (%)	Applied voltage (V)	Ion capacity (mg/g)	Operation time (min)	Ref.
Activated carbon powder	844	51.2	–	–	1.2	0.25	28	[33]
	1491/1594	100	–	–	0.8	0.27	90	[63]
	3073	–	1170	55	1.0	–	10	[110]
	1792			30				
	1501			20				
	984	117	–	96.7	1.2	2.6	72	[39]
	1260	–	200	77.8	1.5	–	180	[36]
	730	–	60	–	1.2	0.13		[93]
			100			6.1		
			200			8		
			500			9.72		
	1153		1000	–	1.2	10.8	180	[111]
			1500			11		
			2000			11.76		
Activated carbon/titania	546	–	500	44.9	1.2	–	200	[47]
Carbon cloth	1500	–	550	–	1.1	10.0	100	[41]
			5500	–	1.1	7.7		
Activated carbon cloth	1980	–	5.85		1	1.75		[94]
Activated carbon cloth/titania	1890				1	4.68		
Activated carbon nanofiber	670	–	4000	–	1.2	8.9	80	[103]
	712	192	–	36.5	1.6	4.64	160	[112]
Activated carbon/ion-exchange resin	–	2000	–	60	1.4	–	18	[35]
Carbon aerogels	400–1100	100	–	–	1.2	3.33	30	[54]
	602	–	140	–	1.2	4.51		[113]
	610	101.6	–	48	1.5	2.81	2400	[114]
				65	1.7	3.76		
	113	–	2000	–	1.3	7.0	300	[115]
OMC	844	51.2	–	–	1.2	0.68	90	[63]
MnO_2_/nanocarbon	558	50	–	81.5	1.2	0.99	50	[46]
	686	50		79.3	1.2	0.95		
Carbon nanotube	153	1500		–	1.2	4.76	200	[116]
		2000				5.24		
Carbon fibre	–	60	–	–	1.2	1.7	100	[117]
		500				2.57		
		1000				3.71		
Carbon nanotube/carbon nanofibre	211	100	–		1.2	3.32	30	[15]
		50			1.2	1.61		
	211	100				3.87	45	[118]
CNT/polyacrylic acid	–	50	–	83	1.2	–	60	[80]
Carbon nanotube sponge	60–80	–	60	–	1.2	4.3	350	[78]
CNT/graphene	435	–	35000	–	1.6	633.3	90	[81]
	479.5	57	–	77	1.0	1.41	120	[82]
	222.1	50	–	–	2.0	1.36	40	[17]
	464	–	250	84.3	2.0	8.6	100	[86]
Functional graphene	406.4	–	65	–	2.0	3.229	30	[84]
Graphene/activated carbon	779	100	–	–	1.2	2.94	100	[48]
Polypyrrole/graphite	0.1407	–	1000	–	1.4	78.73	15	[101]
Activated carbon cloth	1200		100	22 %	1.2	8.5	7	[96]
CNT/micro/mesoporous carbon	526–990		40	98.1	1.2	0.692	120	[79]
Carbon nanofiber	186	89		86	1.2	1.91	90	[102]
CNT	129.2–359.6		5000	95	1		60	[75]
Graphene/mesoporous carbon	685.2	89.5		90	2	0.73,	65	[91]
Graphene/mesoporous carbon spheres	400.4	68.5		80	1.6	2.3	120	[92]
Graphene	384.4		25	88.96	1.2	6.18	90	[89]
Graphene	339	105		65	1.6	2.9	60	[90]
Graphene	220	60		84	1.6	2.256	90	[87]
Three-dimensional hierarchical porous carbon	1036.8		30	92.36	2	2.16	80	[104]

#### Membrane CDI

Conventional CDI is energy inefficient because adsorption and desorption processes happen simultaneously at the EDL. When an electric potential is applied, counter-ions in the pore adsorb onto the electrode and co-ions are expelled from the electrodes which will seriously reduce desalination efficiency. To solve these problems, a membrane capacitive deionization (MCDI) system was introduced by Andelman et al. [105] in which ion-exchange membranes were incorporated along into the electrode fabrication. Including the membranes in the process block co-ions from leaving the electrodes and counter ions can be more fully flushed from the electrode region during ion release procedure increasing ion removal efficiency. Li et al. [106] constructed an MCDI device using carbon nanotube and nanofiber electrodes with ion-exchange membranes. They reported that the salt removal by the MCDI system was about 50 % higher than that by the CDI system. Biesheuvel et al. [107] presented a pilot-plant experimental data for salt removal in MCDI as a function of influent salt concentration and flow rate and found that the salt concentration ratio between the diluted product and the concentrated salt was as high as 25. Choi et al. [44, 108, 109] also developed their own MCDI device has the potential to be very ion-removal efficient. The data shows that salt removal efficiencies of the MCDI cell were enhanced by about 32.8–55.9 % compared to the CDI cell depending on the operating conditions; the current efficiencies were 35.5–43.1 % for the CDI cell but 83.9–91.3 % for the MCDI cell, about twice that of the CDI cell. It was evident that MCDI can increase the desalination efficiency significantly.

### Polyaniline as Conducting Polymer

Polyaniline (PANI) is one of the conducting polymers that has been extensively studied due to its extraordinary electrical properties and good environmental stability [119]. Conducting polymers are mainly organic macromolecular which can be electrically conductive because of the π conjugated double bonds in the polymer back bones. Electrons in the π conjugated double bonds are delocalized and can move along the main chain as charge carrier. Electrical conductivity can also be achieved by movement of holes in occupied energy state. Conducting polymers need to be doped to be conductive or to enhance the conducting property. By doping, it is introducing conductive electrons or holes. When electrons are introduced, it is called n-type doping and p-type doping when holes are introduced.

Polyaniline was first discovered in 1842 in the study of aniline-based dye industry [120]. However, the electrical property and electrochemical property of the substance were not revealed until mid-1980s. MacDiarmid and his group conducted much research on polyaniline, demonstrated the inter conversion of its three oxidation states [121] and elaborated their IR absorption and optical absorption spectroscopy [122]. Also, 15 N NMR [123] and X-ray scattering [124] of the polymer were well studied.

#### Structure of PANI

**Fig. 6:**
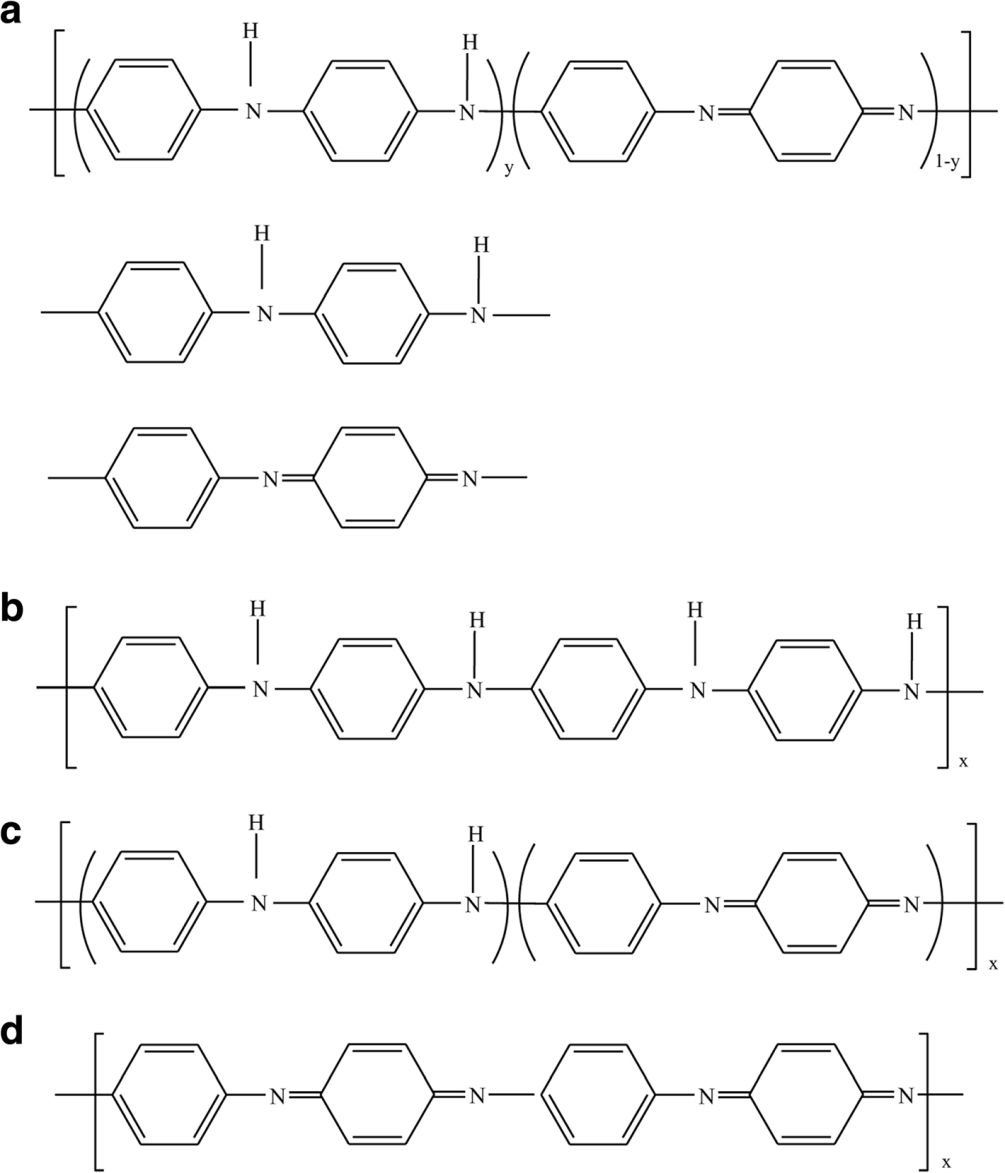
**a** Generalized composition of PANI indicating the reduced and oxidized repeat units. **b** Completely reduced polymer. **c** Half-oxidized polymer. **d** Fully oxidized polymer [126]

**Fig. 7:**
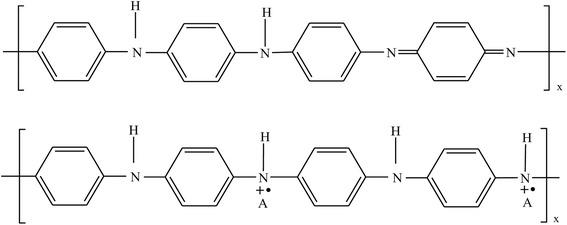
Scheme of proton doping in PANI [127]

PANI has a variety of forms according to its oxidation and protonation state, which exhibit different electrical conductivities and other interesting properties. The base form of PANI consists of alternating quinoid and benzenoid rings with repeat unit chain and can be divided into three states based on the oxidation state: the reduced form (leucoemeraldine), the fully oxidized form (pernigraniline) and the intermediate form (emeraldine). In the reduced form, nitrogen atoms connect entirely with the benzenoid units: in the fully oxidized version, a 1: 1 mixture of benzenoid and quinoid rings; in the emeraldine, with a 3: 1 benzenoid/quinoid ratio (Fig. 6). Treatment of this last state by soaking in acid (see Fig. 7) leads to a dramatic increase in electrical conductivity up to around 500 S/m, and the process is called proton doping [125].

The physical structure of PANI was profoundly revealed along with the study of the conductivity mechanism. The structure was described as a semi-crystalline with order crystalline in the core surrounded by amorphous edge. The doped central crystalline is metallic conductivity in nature, amorphous region has disordered or fold polymer chains which are lower in conductivity and the total conductivity of PANI is restrained by the disordered area [127]. As for which, the total conductivity of PANI depends on the percentage of crystallinity molecular weight molecular arrangement and degree of doping [128]. Mechanism of conductivity was attributed to polaron structure that is formed by nitrogen radicals, which act as charge carrier holes. Except for acids polyaniline could also be n-doped by strong reductant KH or NaH [129]. More recently, carbon nanotubes were proved to be doping agent for PANI [130, 131]. The SWCNT/PANI nanocomposite processed good electrochemical activity in neutral and alkaline media. SWCNTs were considered pristine as potential dopant to preserve the electrochemical property of PANI effectively in the neutral and alkaline media.

#### Polymerization Mechanism and Morphology Controlling

##### Polymerization Mechanism

Chemical polymerization is a conventional method to produce high quality PANI in a large scale. The reaction is initiated by an oxidizing agent such as ammonium persulfate (APS) [132, 133], H_2_O_2_ [134], benzoyl peroxide [135, 136], ferric chloride [137, 138] and chloroaurate acid [139, 140]. APS is considered to be an optical choice due to its high yield.

**Fig. 8:**
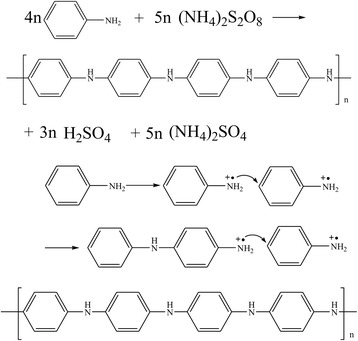
Chemical polymerization of PANI [141]

Chemical polymerization of PANI is shown in Fig. 8. In the beginning, aniline monomers are induced into radical cations by the oxidation agent. The radical cations are recombined into a dimer according to the electrophilic substitution mechanism to start the polymerization. After this induction period, the terminal amine group of oligomer or polymer attacks para-position in monomer causing chain propagation, in which step it is assumed as an electrophilic substitution process [141].

The induction step is reaction. It is observed in experiment that adding aniline dimer accelerates the polymerization significantly [142]. Temperature is an important factor in the aniline polymerization as a low temperature is beneficial for producing high crystalline PANI [143].

##### Morphology Controlling in Chemical Polymerization

Parameters as type of oxidant, acid concentration, reaction temperature and stirring pattern all affect the structure of final polymer. PANI with granular [144], nanotubes/nanowire [145–147], spheres [148, 149] and nanodiscs [150] morphology has been reported depending on the experimental conditions.

**Fig. 9:**
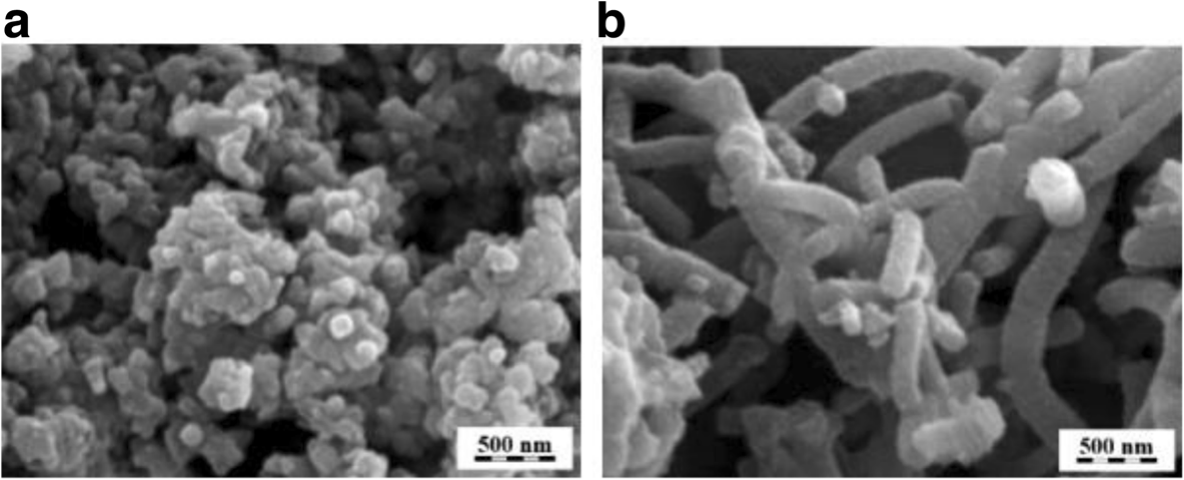
Polyaniline with different morphology (**a** granular **b** nanotubes) [156]

The granular morphology is the most typical form of PANI prepared in acidic aqueous media (see Fig. 9a). This morphology usually forms in high acidic concentration (pH <2.5) and intense monomer concentration (>0.1 mol/L) with a strong oxidant, in which condition the polymer chains grow before the phenazine nucleates organization. So the nucleates packed in random to form globular structure instead of the more organized nanotubes [151]. Nanotubes (Fig. 9b) and nanowires of PANI can be self-assembled directly by polymerization. The methods include diluted monomer [152, 153], weak acid dopant [154–156], interfacial polymerization [157, 158] and rapid mixing [159]. In these conditions, as for the low reactant concentration or low radical cation concentration the growth rate of the backbone is low, the phenazine nucleates are able to stack in ordered cylindrical pattern, producing nanotubes and nanowires. Redox potential of the oxidant is related to the diameter of the grown nanotubes; it was found that the diameter increased with increasing oxidant redox potential [160]. Higher H^+^/aniline ratio result in highly ordered smooth nanotubes with similar diameter [161, 162]. Increase of acid concentration leads to the increase in average diameter of the resultant nanofiber [159].

The ratio of APS to aniline is of importance to the fine surface of nanofibers. Lower APS gives a longer and less branched structure with smaller average diameter [161]. Hollow or filled spherical structures are mostly obtained by emulsion polymerization. In emulsion polymerization, the reaction media composed of oil and water phase. By adding stabilizer and applying appropriate dispersing technique, one phase forms droplets and surrounded by the other phase: oil in water in direct emulsion and water in oil in inverse emulsion. Size of the droplets can be tuned through selection of stabilizer, the ratio of water to oil and the reaction temperature [163].

The two-dimensional disc or plate morphology was also synthesized in acidic aqueous solution with APS as oxidant. The nanodiscs or nanoplates were found to have similar structure in which the nanowires packed together in perpendicular directions to constitute a spiny surface [164]. At high monomer concentration, the dense structure of plat is formed. Controlling the morphology of PANI can also be achieved by seeding polymerization and template synthesis. PANI powder and nanofibres were used as seeds. Nanostructures present during the very early stages of the reaction can orchestrate bulk formation of similar nanostructures. The seeding polymerization is rapid and suitable for bulk quantities production [165]. The synthesized polyaniline nanofibers were found to be high in capacitance. Template synthesis include hard template (i.e. zeolite) and soft template (i.e. water soluble polymers [166], gels [167] and liquid crystalline polymers [168]). In hard template method, the polyaniline grows at the surface of the nanostructured template directly. In soft template, the template forms micelles or guides the polymerization through hydrogen bonding force or electrostatic force [169].

#### PANI as Electrode Material

The electrochemical oxidation and reduction of conducting polymers involves take up and give off ions to maintain electro-neutrality of the material. PANI has three redox peaks which results in large ion capacitance during the ion insertion process and is also attractive with good stability, easy preparation low cost and good conductivity. Since mid-1980s, MacDiarmid and his group demonstrated the inter conversion of its three oxidation states; PANI has been regarded as one of the most promising material for the preparation of rechargeable battery because of this high specific capacitance [121, 128, 170]. In order to use PANI as electrode material in electrochemical battery, intensive research was conducted since 1990s [125, 171, 172].

At first, PANI was electrically synthesized or chemically deposited onto the surface of various substrates, platinum [171, 173], Indium tin oxide [174, 175], graphite [176], etc. The modified electrode showed large redox capacitance. Realizing the porosity and internal microstructure of the electrode material plays a crucial role in ion taking rate as well as capacitance; researchers have been focused on fine structured nano-scope PANI to achieve better property. Several synthetic methods were reported to produce PANI powder [177], nanofibers [137, 146, 152, 153, 160, 178, 179], nanowires [180–184] and nanotubes [133, 147, 156, 185] to exploit high rate and large capacitance.

To optimize the performance of PANI, two approaches were useful. One is to increase specific surface and the other is to enhance electrochemical activity. By producing PANI nanowires, nanofiber and nanotubes specific surface area of PANI increased dramatically. The other is to combine PANI with other materials with high specific capacitance. PANI was combined with polypyrrole nanotubes [186] and nafion nanofibers [187]. It was also combined with electrochemical active metal oxides materials such as MnO_2_ [188, 189], TiO_2_ [190, 191], SnO_2_ [192] and MnWO_4_ [193], for instance. In the meanwhile, combining PANI with large surface materials such as silicon [194], sodium alginate [195], porous carbon, CNT and graphene also proved to be effective approach in increasing the surface area and eventually the capacitance of PANI.

PANI combined with porous carbon to increase the capacitance of carbon and improve the stability of PANI was studied by Chen et al. [196] as electrode material for supercapacitor. With PANI electrochemically deposited onto the surface of porous carbon electrode, the specific capacitance was enhanced and significant contribution of pseudo-capacitance was confirmed. Following the approach, PANI was also combined with various carbon material including porous carbon [197], activated carbon powder [198–200], hollow carbon sphere [201], carbon nanofiber [119, 202] reticulated vitreous carbon [203], calcium carbide derived carbon [204], macroporous carbon [205], mesoporous carbon [206–208] and carbon cloth [209, 210] and carbon black [211]. PANI and activated carbon powder composites were reported have improved capacitance performance compared to bare carbon electrode. PANI-deposited carbon nanofiber electrode was reported to possess high reversibility good stability as well as high specific capacitance. Carbon black acted as cores to form core-shell structures with PANI shell covered uniformly on the surface in which carbon black was considered to be a good component to improve electrical conductivity of the electrode material efficiently. Although incorporating with ordered macroporous carbon resulted in relatively lower specific surface, the PANI composite-based electrode showed high capacitance as good rate capability. The composites of PANI with reticulated vitreous carbon and calcium carbide derived carbon as well as carbon cloth also showed higher specific capacitance than the carbon substrates and good cycle stability. Mesoporous carbon and PANI composites were synthesized though in situ chemical polymerization, the resultant materials were studied and reported to have higher specific capacitance and good reversibility. Furthermore, MnO_2_ was introduced in the PANI/mesoporous carbon composite. The authors concluded that MnO_2_ nanoparticles involved in the material stabilized the interaction between quinoid ring of PANI and OMC/MnO_2_ particles thereby lead to enhance specific capacitance and good cycling stability.

The combination of PANI with carbon nanotubes has drawn great attention since the last decade. Composites of PANI and both SWCNT and MWCNT have been synthesized, and their properties were characterized as electrode materials for supercapacitor. The structural characterization of CNT/PANI composites showed that CNTs were linked up by PANI chains, forming a network structure with new conductive passage and higher conductivity. The strong conjugating interaction between the two components provided fast charge transfer reaction between the two [212, 213]. Capacity tests also showed the composites possessed enhanced specific capacitance. Most of the researchers adopted electrochemical method to obtain CNT/PANI composites as morphology controlling of the composites is convenient in electrochemical polymerization. Zhu et al. [214] fabricated PANI/WMCNT composites by electrochemical approach and found that the specific capacitance of the composites was significantly influenced by its morphological structure and the content of PANI. At the lower, content of PANI was glossily coated onto the tube surface, and the diameter of the composites increased with the increasing PANI content. When the content of PANI reached 76.4 %, the PANI layer became rough and further increasing of PANI content leaded to independently grown PANI nanorods. A maximum specific capacitance of 521.2 F/g when weight percent of PANI was 76.4 % was reported. In accordance, Gupa et al. [215] also reported morphology of electrochemically synthesized PANI/SWCNT composites varied with the content of PANI deposited. As the weight percent of PANI below 70–75 %, the deposited PANI wrapped onto the surface of SWCNT. Beyond the amount, PANI tended to grow independently around the surface or into the micropores. Zhang et al. [216, 217] electrochemically deposited PANI onto the surface of carbon nanotube array in which the nanotube array framework provided large surface area and superconductivity leading to extraordinary energy storage performance. The authors found morphologies of composites were of great importance in improving their performance. Introduction of PANI turned the macro/mesopores of the CNT array into smaller meso/micropores providing higher specific surface area and maintaining regular channels of the material.

In situ chemical polymerization is another well-studied choice to synthesize PANI/CNT composites [218, 219]. Choi et al. [220] chemically synthesize PANI/MWCNT composite in which the surface of MWCNT had been thickly decorated by PANI forming covalent attachment. King et al. [221] investigated the influence of carbon nanotube characteristic on the morphology and properties of the chemically synthesized nanohybrids. It was found that despite aniline monomers themselves could result in nanofibrous structures, CNTs were the dominant templates in the polymerization. However, a less ordered and thinner polyaniline coating was observed at the unoxidized CNTs due to the worse water solubility and weaker hydrogen bonding effects with aniline monomers. By comparing the effects of SWCNT and MWCNT, they found that MWCNT-containing composites was less conducting that the SWCNT-based series and composites based on oxidized CNTs were less conducting than that containing unoxidized ones. More recently, Fan et al. [222] reported chemically synthesized carpenterworm-like PANI/CNT nanocomposites in which MWCNTs were covered by interlace PANI nanoprotuberances. They ascribed the formation of the interesting morphology to the introduction of ethanol in the polymerization solution.

Microwave assisted synthesis [223], liquid-liquid interfacial polymerization [224] and a solid-sate synthesis [225] were reported to obtain PANI/CNT composites. In the former two synthetic methods, PANI coated to the surface of CNTs displaying core-shell structures. In solid state synthesis, the composite containing 16 % MWCNT was aggregated particles in morphology while the resultant containing 32 % MWCNT showed some nanofiber morphology. Combination of PANI/CNT and transition metal oxidize MnO2 was also studied to further improve the capacitance performance [226, 227].

Dong et al. [228] studied electrochemical performance of PANI/MWCNT composites in neutral aqueous solution and found that the composites showed higher specific capacitance than pure PANI. Infrared spectroscopy studies have shown that introduction of CNTs affected both the free N–H environment and quinoid units in PANI backbones indicating doping effect of CNTs [130]. Zhou et al.’s [131] study revealed that mixture of SWCNT and PANI processed good electrochemical activity in neutral and alkaline media. The authors concluded that fast electron transfer property and high stability of the mixture in neutral and alkaline media were due to effective doping of PANI by SWCNT.

More recently, due to arising of different methods of graphene preparation, the unique electrical property of graphene and promising electrochemical characteristics of PANI, combination of the two draw great attention. Studies in the field covered both the combination of graphene oxide and graphene with PANI. Methods of fabrication included in situ polymerization of aniline [229–232], filtration of the mixed dispersion [233, 234] and grafting PANI nanofibers to the edge of graphene sheets [235, 236] as well as layer-by-layer self-assemble method [237]. Further studies use functionalized graphene to composite with PANI in order to obtain higher capacitance. Li et al. [229] synthesized sandwich-like graphene/PANI composite in which PANI particles covered homogeneously on the surface of the graphene sheets at lower aniline ratio. Increasing the aniline ratio leaded to larger particle size of the deposited PANI. When the aniline ratio exceeded 12:1, fibrous PANI grew simultaneously with the sandwich composite. In situ polymerization can also produce graphene/PANI nanofiber composite in which nanofibers stick paralleled onto graphene sheets [231]. PANI was reported to anchor onto partially reduced graphene sheets and form a loose flower-like structure that extended the specific surface area of the material [238]. Polymerization of PANI in the presence of graphene oxide could result in the direct exfoliation of GO. PANI grew into layered GO causing peeling of the GO layers forming a single-layered GO/PANI nanosheet with folded edge [239]. Wu et al. [234] developed supercapacitor based on paper-like flexible graphene/PANI nanofiber composite films. The films obtained by filtrating mixed dispersions of graphene nanosheets and PANI nanofibers showing high flexibility that could be bent into large angles. By directly filtration of an in situ polymerized, a mixture of PANI and GO and a mixture of reduced GO, Yan et al. [233] obtained flexible composites paper and found that compactable small particles were formed on the GO sheets while nanorods and large particles were formed on the reduced GO substrate. The authors assumed that nanorods came from the individual PANI fibres formed in the aqueous phase, but why PANI fibres adsorbed onto graphene not GO was not explained. A comparison study on surface functionalities of GO with N and NH_2_ groups was carried out by Lai et al. [240]. It was found NH_2_-functionalized GO/PANI (NH_2_-GO/PANI) showed high conductive stable composite. Interestingly, an increase in capacitance was observed for NH_2_-GO/PANI composite. The authors suspected that the amine functional groups assisted the doping and dedoping in PANI causing the enhancement of capacitance in long cycles. Others [235, 236] tried grafting PANI nanofibers to the edge of graphene sheets through an amide-connecting system. The PANI nanofiber connected to the graphene intimately and distributed homogeneously along of graphene layers inhabiting the agglomeration of the layers. Computing of the electronic densities of the system showed that π-π conjugation was extended from graphene to PANI chains through the amide group. O=C–N group also played as electron bridge spreading the electrons along the conjugation system. The conjugation system facilitated the fast transfer of electron.

Multilayered PANI/GO films could be built up on top of silica or ITO (indium tin oxide) glass through layer-by-layer self-assemble. Specifically, the substrate was immersed alternatively into the positive-charged PANI solution and the negative-charged GO dispersion forming compact stacked bilayers, and the GO could be reduced into graphene resulting in graphene/PANI films. More importantly, the graphene/PANI films demonstrated good electrical conductivity and high volumetric capacitance even in a neutral Na_2_SO_4_ electrolyte [237].

Dong et al. [241, 242] grew 3D graphene on the substrate of nickel foams and chemically synthesized PANI onto the surface of the 3D feature obtaining porous graphene/PANI composite which exhibited high specific capacitance and good cycling stability. A controlled morphology of hierarchical PANI nanowire arrays grown on the plane of GO nanosheets was obtained by Xu et al. [182]. The authors suspected the oxygen functional groups on the plane of the GO sheets acted as anchor sites and enabled the attaching of PANI nanowires on the surface of GO. The π-π stacking force was also considered to be the beneficial factor that promoted the initial nuclei of PANI on the GO sheets. Later, Li et al. [243] synthesized oriented PANI arrays on expended graphite sheets. The morphology of the composites could be adjusted by varying the ratio of expended graphite sheets and aniline monomer. These methods demonstrated morphology engineering at the nanoscale level, producing convenient approaches for fabricating conducting polymer composite material with designed morphology.

In various GO or graphene and PANI composites, the capacitance were greatly improve by introducing PANI along with high cycling stability due to the synergistic effect between the two components (Table 2). The close contact of graphene sheets and PANI backbone allows strong conjugation interaction between the two that promotes fast charge transfer resulting in improved electrochemical properties. Also, the fast charge transfer prevents the charge accumulation of electrons produced by faradic reaction of PANI thus decelerating structure conformation of PANI during electrochemical reaction which eventually benefit to good cycling stability. Moreover, with careful engineering, PANI could modify pore structure of graphene by acting as a spacer to stop layer restack to produce favourable pores facilitating the transfer of ions within the electrolyte.Composites based on graphene/CNT/PANI were synthesized and studied as electrode material for supercapacitors. The composite showed high specific capacitance comparable to or even better than that of graphene/PANI composites [244]. More importantly, the graphene/CNT/PANI showed a super high cycling retention of 96 % after 5000 cycles [245].

**Table 2:** Capacitance performances of PANI and PANI composites

Electrode materials	Synthetic methods	Specific capacitance (F/g)	Capacitance retention	Test electrolyte	Published year	Ref
PANI powder	Chemical polymerization	107	79 % after 9000 cycles	1 M EtNBF_4_	2002	[177]
PANI nanowires	Electropolymerization	775	91 % after 1000 cycles	1 M H_2_SO_4_	2006	[246]
PANI nanofibers	Chemical polymerization	428	–	1 M H_2_SO_4_	2008	[179]
PANI nanowires	Electropolymerization	700 at 5 A/g	–	1 M H_2_SO_4_	2008	[180]
PANI nanowires	Electropolymerization	1142 at 5 A/g	95 % after 500 cycles	2 M H_2_SO_4_	2008	[170]
PANI nanofibers	Electropolymerization	480 at 5 mV/cm^2^	–	1 M KCl and 10^-3^ M HCl	2009	[247]
PANI nanobelts	Electropolymerization	873	96 % after 500 cycles	1 M H_2_SO_4_	2010	[248]
PANI nanofibers	Electropolymerization	839	95 % after 500 cycles	1 M H_2_SO_4_	2010	[249]
PANI nanowires	Electropolymerization	950 at 1 A/g	80 % after 500 cycles	1 M HClO_4_, 1 M LiTFSI	2010	[181]
Nanostructured PANI	Chemical bath deposition	503	–	1 M H_2_SO_4_	2011	[250]
PANI nanowires	Electropolymerization	882	95 % after 500 cycles	0.5 M H_2_SO_4_	2013	[183]
Polypyrrole/PANI	In situ polymerization	416	–	1 M H_2_SO_4_	2008	[186]
Nafion/PANI	Solution cast	235	84 % after 10000 cycles	1 M H_2_SO_4_	2010	[187]
MnO_2_/PANI	Static adsorption	330 at 1 A/g	94 % after 1000 cycles	0.1 M Na_2_SO_4_	2007	[188]
MnO_2_/PANI	In situ polymerization	510 at 1 A/g	–	0.5 M Na_2_SO_4_	2010	[189]
TiO_2_/PANI	In situ polymerization	330 at 1.5 A/g	122 % after 3000 cycles and 92 % after 10000 cycles	1 M H_2_SO_4_	2009	[190]
TiO_2_/PANI	In situ polymerization	784	–	0.5 M H_2_SO_4_	2012	[191]
SnO_2_/PANI	In situ polymerization	325 at 30 A/g	–	1 M H_2_SO_4_	2012	[192]
MnWO_4_/PANI	In situ polymerization	481 at 18 A/g	–	1 M H_2_SO_4_	2012	[193]
Silicon/PANI	Electropolymerization	470 at 5 mV/cm^2^	78 % after 700 cycles	0.5 M H_2_SO_4_	2010	[194]
Sodium alginate	In situ polymerization	2093	74 % after 1000 cycles	1 M H_2_SO_4_	2011	[195]
Porous carbon/PANI	Electropolymerization	180	90 % after 1000 cycles	1 M H_2_SO_4_	2003	[196]
Activated carbon/PANI	Electropolymerization	270	–	1 M H_2_SO_4_	2004	[198]
Activated carbon/PANI	Electropolymerization	587	–	0.5 M H_2_SO_4_	2008	[199]
Activated carbon/PANI	In situ polymerization	956	–	6 M KOH	2011	[200]
Hollow carbon sphere	In situ polymerization	525 at 0.1 A/g	73 % after 1000 cycles	2 M H_2_SO_4_	2010	[201]
Carbon nanofiber/PANI	Vapour deposition polymerization	264	–	1 M H_2_SO_4_	2005	[119]
Carbon nanofiber/PANI	In situ polymerization	638 at 2 A/g	91 % after 1000 cycles	1 M H_2_SO_4_	2011	[202]
Calcium carbide-derived carbon/PANI	In situ polymerization	713	80 % after 1000 cycles	1 M H_2_SO_4_	2010	[204]
Mesoporous carbon/PANI	In situ polymerization	87.4 at 5 mA/cm^2^	90 % after 1000 cycles	1 M H_2_SO_4_	2010	[206]
OMC/PANI	In situ polymerization	409 at 0.1 A/g	–	30 wt.% KOH	2011	[207]
OMC/PANI	In situ polymerization	400 at 1 A/g	80 % after 1000 cycles	6 M KOH	2011	[208]
Carboncloth/PANI	Electropolymerization	673	–	1 M H_2_SO_4_	2011	[210]
Carboncloth/PANI	Electropolymerization	408 at 1 A/g	30 % after 1000 cycles	0.5 M Na_2_SO_4_	2012	[209]
Carbon black/PANI	layer-by-layer assembly	532 at 10 mA/cm^2^	–	1 M H_2_SO_4_	2013	[211]
PANI/mesoporous carbon/MnO_2_	In situ polymerization	695 1 A/g	88 % after 1000 cycles	1 M H_2_SO_4_	2012	[251]
Carbonized PANI nanotubes	Chemical polymerization and carbonization	165 at 0.1 A/g	–	30 wt.% KOH	2010	[252]
SWCNT/PANI	In situ polymerization	191 at 0.25 A/g	–	1 M NaNO_3_	2004	[219]
SWCNT/PANI	Electropolymerization	463 at 10 mA/cm^2^	95 % after 500 cycles	1 M H_2_SO_4_	2006	[215]
CNT/PANI	In situ polymerization	350 at 1 A/g	92 % after 1000 cycles	0.5 M H_2_SO_4_	2010	[253, 254]
SWCNT/PANI	Electropolymerization	1000	–	0.5 M H_2_SO_4_	2011	[255]
MWCNT/PANI	Chemical vapour deposition	328 at 5 mA/cm^2^	94 % after 1000 cycles	1 M NaNO_3_	2007	[228]
MWCNT/PANI	In situ polymerization	322 at 1 mA/cm^2^	–	1 M H_2_SO_4_	2007	[223]
MWCNT/PANI	In situ polymerization	606 at 1 A/g	64 % after 1000 cycles	1 M H_2_SO_4_	2007	[256]
CNT array/PANI	Electropolymerization	1030 at 5.9 A/g	>94 % after 5000 cycles	1 M H_2_SO_4_	2008	[202]
MWCNT/PANI	Electropolymerization	500 at 5 mA/cm^2^	68 % after 1000 cycles	0.5 M H_2_SO_4_	2009	[257]
MWCNT/PANI	In situ polymerization	238 at 0.25 A/g	–	1 M H_2_SO_4_	2010	[258]
MWCNT/PANI	In situ polymerization	560	71 % after 1000 cycles	0.1 M H_2_SO_4_	2010	[213]
MWCNT/PANI	Solid-state polymerization	522 at 3 mA/cm^2^	–	1 M H_2_SO_4_	2011	[225]
MWCNT/PANI	In situ polymerization	250	83 % after 100 cycles	0.1 M H_2_SO_4_	2011	[220]
CNT/PANI	In situ polymerization	440 at 1 A/g	96 % after 1000 cycles	1 M H_2_SO_4_	2012	[259]
MWCNT/Sulphur/PANI	In situ polymerization	1334 mAh/g	70 % after 80 cycles	1 M LiTFSI in DOL:DME (1:1, *v*/*v*)	2011	[218]
MWCNT/PANI/MnO_2_	In situ polymerization	330	77 % after 1000 cycles	0.5 M Na_2_NO_3_	2011	[227]
GO/PANI	In situ polymerization	746 at 0.2 A/g	73 % after 500 cycles	1 M H_2_SO_4_	2010	[260]
GO/PANI nanoarrays	In situ polymerization	555 at 0.2 A/g	92 % after 1000 cycles	1 M H_2_SO_4_	2010	[182]
Partially reduced GO/PANI	In situ polymerization	330 at 5 mA/cm^2^	~87 % after 1000 cycles	1 M H_2_SO_4_	2013	[238]
Graphene/PANI	In situ polymerization	408	84 % after 40 cycles	1 M H_2_SO_4_	2009	[232]
Graphene/PANI	Electropolymerization	233	Slightly increase with longer cycles	1 M H_2_SO_4_	2009	[261]
Graphene/PANI nanofiber	In situ polymerization	480 at 0.1 A/g	–	2 M H_2_SO_4_	2010	[262]
GO/PANI	In situ polymerization	320 at 0.1 A/g	67 % after 5 cycles			
Graphene/PANI	In situ polymerization	1126	84 % after 1000 cycles	1 M H_2_SO_4_	2010	[263]
Graphene/PANI	In situ polymerization	1046 at 1 mV/s	67 % after 1000 cycles	6 M KOH	2010	[245]
Graphene/PANI	In situ polymerization	489 at 0.4 A/g	>96 % after 500 cycles	1 M H_2_SO_4_	2010	[233]
GO/PANI		366 at 0.4 A/g	–			
Graphene/PANI	In situ polymerization	450	~90 % after 1000 cycles	1 M H_2_SO_4_	2011	[264]
Graphene/PANI	Adsorption	301 0.5 A/g	67 % after 1000 cycles	1 M H_2_SO_4_	2011	[265]
Graphene/PANI	In situ polymerization	699	92.8 % after 1000 cycles	1 M H_2_SO_4_	2012	[229]
Graphene/PANI	In situ polymerization	846	79.4 % after 1000 cycles			
Graphene /PANI nanofiber	In situ polymerization	526 at 0.2 A/g	–	2 M H_2_SO_4_	2012	[266]
Functionalized graphene/PANI	In situ polymerization	355 at 10 A/g	–	1 M H_2_SO_4_	2011	[267]
Graphene/PANI	In situ polymerization	1130	87 % after 1000 cycles	1 M H_2_SO_4_	2011	[231]
NH_2_-RGO/PANI	In situ polymerization	500	119 % after 680 cycles and then decrease	1 M H_2_SO_4_	2012	[240]
Graphene/PANI	In situ polymerization	361 at 0.3 A/g	~82 % after 1000 cycles	–	2012	[264, 268]
Graphene/PANI	In situ polymerization	250	–	1 M H_2_SO_4_	2012	[230]
Graphene/PANI nanofiber	Filtration	210 at 0.3 A/g	71 % after 800 cycles	1 M H_2_SO_4_	2010	[234]
Graphene/PANI nanofiber	Nanofiber grafting	623 at 0.3 A/g	82 % after uncertain cycles	2 M H_2_SO_4_	2012	[235]
Graphene/PANI nanofiber	Nanofiber grafting	580 at 0.3 A/g	96 % after 200 cycles	2 M H_2_SO_4_	2012	[236]
Graphene/PANI	Layer-by-layer assembled films	584 F/cm^3^ at 3 A/cm^3^	~56 % after 1000 cycles	1 M Na_2_SO_4_	2012	[237]
Graphene/PANI oriented arrays	In situ polymerization	1665 at 1 A/g	97 % after 2000 cycles	1 M H_2_SO_4_	2013	[243]
3D graphene/PANI	In situ polymerization	346 at 4 A/g	71 % after 120 cycles	1 M H_2_SO_4_	2012	[241]
Graphene/PANI/CNT	In situ polymerization	1035	94 % after 1000 cycles	6 M KOH	2010	[245]
Graphene/PANI/CNT	Vacuum filtration	569 at 0.1 A/g	96 % after 5000 cycles	1 M HCl	2011	[244]
GO/activated carbon cloth/PANI	Electropolymerization	369 at 50 mA/g	80 % after 1000 cycles	1 M H_2_SO_4_	2012	[269]

## Conclusions

CDI is a novel ion removal technology that uses static electrical force to drive ions to the charged electrode and stores the ions into porous structure of the electrode. Developing more efficient electrode materials is the key to improving salt removal performance. The current work reviewed up-to-date progress on electrode fabrication in application of CDI. Fundamental principal (e.g. EDL theory and adsorption isotherms) and process factors (e.g. pore distribution, potential, salt type and concentration) of CDI performance were presented first. It was then followed by in-depth discussion and comparison on properties and fabrication technique of different electrodes, including carbon aerogel, activated carbon, carbon nanotubes, graphene and ordered mesoporous carbon. Novel nanomaterials such as graphene and CNTs have been drawing increased attention in this field with their extremely high specific surface area and super electrical properties. However, research in the use of CNT and graphene as CDI electrodes is still in its infancy, and initial milestones such as large scale of synthesis and fabrication of high surface area graphene and CNT CDI electrodes have not been reached yet. Although CDI and MCDI have shown practicality and cost-effectiveness in brackish water treatment, only few semi-pilot scale demonstration of the CDI or MCDI has been performed so far and no complete pilot scale demonstration has been performed yet [270–272], not to mention demonstrations of graphene and CNT-based CDI systems. Full-scale CDI design with enhanced energy efficiency will be the focus of future endeavour in this area. Finally, it is noteworthy that PANI as conductive polymer has great potential in the CDI application as electrode-enhancing materials. It is envisaged that large-scale PANI-assisted CDI electrode fabrication can be plausible due to its low cost, mechanical flexibility and good electrical properties.
